# Heritage Conservation Future: Where We Stand, Challenges Ahead, and a Paradigm Shift

**DOI:** 10.1002/gch2.202100084

**Published:** 2021-10-15

**Authors:** Jorge Otero

**Affiliations:** ^1^ Department of Mineralogy and Petrology University of Granada Fuentenueva s/n Granada 18002 Spain

**Keywords:** commons, global challenges, heritage conservation, open‐science, peer‐to‐peer, professional activism, social movements

## Abstract

Global cultural heritage is a lucrative asset. It is an important industry generating millions of jobs and billions of euros in revenue yearly. However, despite the tremendous economic and socio‐cultural benefits, little attention is usually paid to its conservation and to developing innovative big‐picture strategies to modernize its professional field. This perspective aims to compile some of the relevant current global needs to explore alternative ways for shaping future steps associated with the 2030 Agenda for Sustainable Development. From this perspective, it is conceptualized how emerging artificial intelligence (AI) and digital socio‐technological models of production based on democratic Peer‐2‐Peer (P2P) interactions can represent an alternative transformative solution by going beyond the current global communication and technical limitations in the heritage conservation community, while also providing novel digital tools to conservation practitioners, which can truly revolutionize the conservation decision‐making process and improve global conservation standards.

## Graphical abstract

Cultural heritage is a lucrative asset. However, despite its tremendous worldwide economic and socio‐cultural benefits, little attention is usually paid to reflect on novel big‐picture strategies to modernize the conservation field. This perspective reviews some of the relevant current global challenges and conceptualizes how emerging digital‐social‐movements based on Peer‐2‐Peer interactions can represent a truly transformative solution to go beyond current needs.

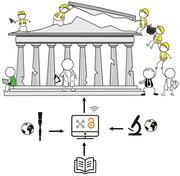

## 1 Introduction

Cultural heritage refers to the legacy of tangible items (i.e., buildings, monuments, landscapes, books, textiles, paintings, or archaeological artifacts) and their intangible attributes (i.e., folklore, traditions, language, or performance arts) that are inherited from the past by a group or society and conserved for future generations due to their artistic, cultural, or historic value.^[^ ^1^ ^]^ The act of preserving cultural heritage is known as Heritage Conservation, and it mostly focuses on doing everything possible to delay the natural laws of deterioration on tangible items to guarantee the transmission of its significant heritage messages and values for future generations. Current heritage conservation practice activities, which are mostly carried out by conservation practitioners (i.e., conservators–restorers and conservation technicians) in worldwide museums, conservation laboratories and monuments; widely involve activities such as the implementation of preventive actions (i.e., controlling the surrounding environmental conditions of items to mitigate damage), remedial activities (i.e., applying a conservation treatment to strengthen item's properties) or the application of a restoration process to bring decayed items as nearly as possible to their former condition. Conservation scientific research activities, which are mostly carried out by conservation scientists in worldwide universities and heritage research institutions, support the conservation practice providing scientific advances in the characterization of materials, the investigation of the material's degradation phenomena and the development of materials and technologies for their conservation and restoration.^[^ ^2^ ^]^

Cultural heritage represents nowadays one of the most important global industries and a substantial economic benefit for host countries, regions, and local communities. According to the latest studies made by the World Travel and Tourism Council, in 2019, cultural tourism represented 40% of all European tourism, generating 319 million jobs and producing more than 30 billion € in revenues every year.^[^ ^3^ ^]^ Besides the economic asset and tourist attraction, cultural heritage also has a significant value as an identity factor contributing to social cohesion.^[^ ^4^ ^]^ Despite the tremendous economic and socio‐cultural benefits, little attention and investment are usually taken on its conservation and/or to develop new strategies to modernize its practice activities. Machu Picchu, Taj Mahal, Petra or Angkor, among many other monuments with irreplaceable cultural heritage significance, are currently eroding at a noticeable rate^[^ ^5^, ^6^, ^7^, ^8^ ^]^ and current global conservation activities are not completely succeeding in the implementation of quality conservation strategies to stop damage.^[^ ^9^ ^]^ According to the latest *heritage at risk* report made by ICOMOS in 2020,^[^ ^10^ ^]^ ≈65% of the world's buildings with artistic and/or cultural interest currently present lack of maintenance and are in a poor state of conservation, which leads structures to a constant loss of its cultural, artistic, and economic value. Such loss has drawn recently the attention of the international political community, which has recognized the need to safeguard this heritage, as represented by one of the 169 specific targets of the Sustainable Development Goals (SDG 11.4). Inadequate environmental conditions, climate change, the massification of tourism, and insufficient management and resources are nowadays the major conservation threats to World Heritage Sites.^[^ ^11^ ^]^ Considering that the cultural tourism industry has been globally growing, at a rate of 20–25% in the last 10 years before the COVID‐19 pandemic eruption,^[^ ^12^ ^]^ added to the effect of global warming and the current high levels of pollution in urban areas, the decay of heritage items is expected to increase considerably in the next 10 years.^[^ ^13^, ^14^, ^15^, ^16^, ^17^ ^]^ This rapid deterioration is expected to be even more exacerbated in developing countries since conservation activities are often carried out by inexpert and/or untrained practitioners^[^ ^18^ ^]^ which, in several cases, can increase damage up to 50%.^[^ ^19^ ^]^ In this context, there is a pressing need to envision innovative solutions to develop different global strategies to go beyond the current global challenges in the heritage conservation community for better conservation outcomes and to continue enjoying the tremendous economic benefits derived from heritage more efficiently and sustainably for the benefit of global future generations.

On the other hand, cultural heritage conservation can also serve as a worldwide economic driving force, but especially in economically and socially marginalized communities in developing countries since it helps to generate local jobs, creation of opportunities for income‐generation and jobs (especially for youth and women), better learning opportunities for all, reducing inequality between social status or communities, improving professional competitiveness in skilled jobs and promoting cooperation between stakeholders and professional entities, increase tourism, and improve the quality visitor experience.^[^ ^20^ ^]^ Besides the economic growth in developing countries, cultural heritage conservation enables sustainable development by enhancing the inhabitants’ sense of identity, feeling of connection, and improves people's well‐being.^[^ ^21^ ^]^

This communication aims to assemble some of the current global challenges in heritage conservation and propose an alternative paradigm for shaping up future steps associated with the 2030 Agenda for Sustainable Development.

## 2 Global Challenges Ahead in Heritage Conservation

### 2.1 Analysis of the Heritage Conservation Scientific Data

The ability to uncover insights and trends in large amounts of data has been around since ancient times. Ancient Egyptians used the analysis of data to increase efficiency in tax collection or accurately predict the flooding of the river Nile every year.^[^ ^22^ ^]^ However, data science, or “big data analysis,” has especially emerged in the last decade as a key new area of study having a tremendous impact in other scientific areas such as biology, medicine, or the development of smart‐green cities, which is able to extract new value from large complex unstructured data coming from differences sources.^[^ ^23^, ^24^, ^25^ ^]^ The interest to study heritage materials is an old field of research, which started back in the XIX century where scientists such as Michael Faraday (1791–1867),^[^ ^26^ ^]^ Friedrich W. Rathgen (1862–1942),^[^ ^27^ ^]^ or A. W. von Hofmann (1818–1892)^[^ ^28^ ^]^ had already drawn the attention to the study of the degradation phenomenon of heritage materials. However, to date, there has not been a single work on any macroperspective analysis or data science applied to the understanding and management of the conservation data from heritage. This is surprising especially for three reasons: i) studies of the heritage conservation are incredibly data‐rich and spread in a vast number of sources; ii) current research is still progressing without macroperspective directions; iii) most excellent scientific findings lack nowadays the adequate dissemination and are rarely transferred into practice.

I believe that, at this point, heritage conservation data requires the appropriate analysis in order to derive meaningful information crucial to help scientists and conservation research institutions to find new key areas of research and optimize research activities. At this point, should the emphasis of heritage conservation be placed on the development of new materials and new application procedures? Are most of the damage mechanisms already precisely understood and linked to visible decay patterns? Has there been significant uncover work that needs to be transferred to real practice? Have similar studies obtained similar results? Are the techniques and methods for evaluating heritage materials and decay processes accessible to conservation practitioners and is this methodology universally accepted by the scientific community? Can this methodology and findings be implemented by conservation practitioners also in developing countries? Does science need to provide more research to evaluate the long‐term durability of treatments? etc. In this light, I believe there is an urgent need to analyze the existing scientific data before continuing with more incremental research data to evaluate the direction in which research has been progressing and whether or not the current direction is proving fruitful.

But, how can we tackle such complex and macroscopical analysis? Big data technologies (software and data warehouse), together with the increased use of cloud‐based, high‐performance computing (algorithms), and artificial intelligence (AI), can create new opportunities for data analysis with tremendous benefits to any multidisciplinary and data‐rich fields as health,^[^ ^29^ ^]^ history,^[^ ^30^ ^]^ or even heritage conservation.^[^ ^31^ ^]^ However, although these big data technologies could be very useful to extract unknown correlations, detect hidden patterns, detect areas of overproduction, areas that lack research or help us to obtain similarities or differences on similar projects,^[^ ^32^ ^]^ those algorithms have currently difficulties to establishing qualitative analyses to highlight crucial findings, which can help us answer the mentioned questions; especially considering diverse and complex environments,^[^ ^33^, ^34^, ^35^, ^36^ ^]^ such as the conservation of cultural heritage, which requires frequently the consensus/input of professionals with very different angles based on diverse expertise, context, and environments. Moreover, considering that the big data analysis is usually carried out by only one researcher or by a selected group of experts, this analysis has been found to be highly unconsciously biased by the researcher's previous experience/scientific position and often this big data analysis is not unanimously accepted on multidisciplinary environments involving different fields, academic positions, and research interests.^[^ ^37^, ^38^, ^39^ ^]^ So, how can we provide the first step to create a summary of the conservation existing scientific findings that could be accepted consensually by both its scientific and practice community?

### 2.2 Reduce Inequalities: Bridge the Gap between Developed and Developing Countries

Scientific journals are still nowadays the principal channel for disseminating research results across the global scientific community. However, access to those scientific journals is highly expensive and also restricted to some developing countries, which is called by UNESCO “the information gap.”^[^ ^40^ ^]^ In the developed world, the majority of research institutions and universities provide their scientists with unlimited updated online access to most scientific journals.^[^ ^41^ ^]^ However, in developing countries, where most conservation is needed, most research institutions cannot afford them and scientists suffer from a serious lack of access to advanced and up‐to‐date peer‐reviewed scholarly literature.^[^ ^42^ ^]^ A World Health Organization (WHO) survey conducted in 2000^[^ ^43^ ^]^ reported that ≈65% of research institutions in developing countries have no subscription to any international scientific journals. Another relevant survey published in Nature^[^ ^44^ ^]^ revealed that only eight nations in the world produce 85% of total publications globally. Unfortunately, this isolation is unconsciously promoted by developed‐country scientists who are usually encouraged and expected to publish research in “high profile” journals to increase competitiveness. This, in turn, facilitates access to further research funding, but this also further accentuates the information gap between developed and developing countries. If such asymmetry in research output and access to up‐to‐date information remains a characteristic of the scientific world, then conservation practitioners and scientists in developing countries will remain isolated and their work will continue to have an important lack of updated technical expertise, which will affect directly the conservation of their cultural heritage. In this light, further initiatives in conservation should aim, as much as possible, to promote open‐science and provide a better, wider, and more equal access to knowledge.

### 2.3 Increase the Synergetic Exchange of Knowledge between Science and Practice: Promoting Interdisciplinary

It is widely accepted within the heritage conservation community that there is a considerable gap between science and practice. Closing this gap has been the theme of several conferences, books and international debates.^[^ ^45^, ^46^, ^47^ ^]^ There are many reasons why this gap exists. First, a high number of papers published by conservation scientists in scientific journals are seldom read outside of the academic world and there are few incentives for researchers to bring their science into practice. On the other hand, conservation practitioners rarely publish and/or document any of their field/hands‐on experiences and experiments in a manner that can meaningfully inform conservation scientists. Other reasons, such as the lack of access to scientific literature (high cost of journals, as previously mentioned), the fact that each field has different professional goals and the limited relevance of conservation practitioners in the decision‐making process within heritage multidisciplinary projects, are factors that really exacerbate the divide. This is obviously added to a fear of a critical analysis at all levels of the conservation theory and practice by both sides. Additionally, since conservation science is a relatively new discipline, most conservation scientists are trained in one of the natural sciences (e.g., Physic, chemistry or engineering) who specialized in heritage conservation directly through employment or personal interest in cultural heritage.^[^ ^48^ ^]^ They publish most of their findings in scientific Journals specialized in other disciplines, where practitioners have usually no connection to them and/or have no technical knowledge to correctly extract the information they need from them. During the last decade, new digital professional networks (mostly LinkedIn, Academia, and Research gate) have improved interdisciplinary global interactions between conservation peers, and are currently used as the main digital communication medium between heritage conservation professionals outside main international heritage organizations (i.e., ICON: Institute for Conservation; AIC: American Institute for Conservation; ECCO: European Confederation of Conservator‐Restorers’ Organizations; ENCORE: European Network for Conservation‐Restoration Education; ICOM‐CC: International Council of Museums; IIC: International Institute for Conservation; and ICOMOS: International Council on Monuments and Sites). However, although those networks are very effective platforms to share new research and new published experiences, neither of them allows high levels of user's interaction in order to create discussion/dialogue on research outputs or consensually organize and summarize findings to create new knowledge. Additionally, they barely allow documenting any unpublished experiences of remarkable observations obtained by practitioners (or scientists) on their hands‐on experience in a manner that can be useful to other heritage professionals. In this context, it is clear that new strategies are needed to create a greater synergy between science and practice.

### 2.4 Document, Transmit, and Preserve the Current Knowledge Contained in Practice Activities

Conservation practice activities carried out by practitioners are highly observational, “knowledge gained by experience” and require a high level of manual dexterity for the use of tools and analytical methods. Furthermore, success or failure in interventions is often highly influenced by the practitioner's skill and experience.^[^ ^49^, ^50^, ^51^ ^]^ In several cases, conservation practice activities are traditional methods passed from generation to generation, such as the Mughal‐era tile conservation method in India^[^ ^52^ ^]^ or earthen architectural conservation skills in Mali,^[^ ^53^ ^]^ which present the serious risk to disappear without being properly documented.^[^ ^54^ ^]^ In this context, global conservation online forums within international heritage organizations currently provide the main communication vehicle where practitioners can organize in professional groups based on their expertise to share knowledge and create discussion on specific topics. However, although those conventional digital forums are effective as a knowledge‐sharing vehicle, this conventional way of professional interactions do not allow users to document, organize into categories, and summarize experience and knowledge that can create new added value. This is added to other factors such as the lack of open‐access accessibility to those forums (membership) and that rarely contemplate accessible video tutorials to stimulate training for other professionals.

### 2.5 Create a More Participatory System to Understand and Disseminate the Current Scientific Knowledge

“Dissemination of research ensures that research communities are able to build on existing knowledge, highlight new discoveries, and do not duplicate efforts in either research or implementation” (UNESCO, 2008).^[^ ^55^ ^]^ The amount of currently available heritage scientific data is overwhelming. The conservation science field, as in other scientific fields, went from a significant lack of data to a data deluge in just 30 years.^[^ ^56^ ^]^ Large heritage research databases exist (e.g., AATA, JTOR, or ICOMOS library) at different scales, but can conservation scientists efficiently track this large amount of unstructured new data? And, are the most remarkable findings really reaching the practitioners? In reality, few researches are properly disseminated beyond academia to make a real impact in the practical field. However, even when research is accordingly disseminated through professional platforms (e.g., Research‐gate or Academia), indexed in online repositories (e.g., Scopus or Web of Science), presented in heritage recognized conferences (e.g., ICOM‐CC or IIC) and included in heritage digital libraries, due to the high complexity of the conservation field, it is difficult to establish reliable comparisons among current research and data. I have to constantly face this complexity in my professional scientific field. For example, one of my current research interests studies the consolidation effectiveness of nanolime when applied to a historic structure. However, its effectiveness has been discovered to be influenced by many factors such as its concentration (g L^−1^), solvent, application method, amount of product applied, application procedure, crystallinity, size and surface area of the nanolime particle, type of substrate, product storage time, pore size distribution, and mineralogical composition of the substrate or relative humidity conditions during the curing time.^[^ ^57^, ^58^, ^59^, ^60^, ^61^ ^]^ This wide range of variables makes it extremely difficult to draw accurate and reliable comparisons among current research findings, which often requires a personal communication (e.g., videoconference meetings, phone calls, or emails) between involved scientists to draw common conclusions on specific topics. Conventional mentioned e‐libraries and networks allow the visualization of our new research findings, but do not allow us high levels of user's coordination in order to discuss, compare, and classify while building on a commonly agreed shared knowledge for the benefit of other scientists and the practice.

### 2.6 Assist Global Practitioners with Tools to Enhance Their Conservation Activities

According to a well‐known work carried out by the heritage architect J. Fidler in 2005,^[^ ^18^ ^]^ about 60% of global conservation and maintenance activities are nowadays carried out by inexpert and/or untrained practitioners, which in several cases, can increase damage up to 50%, especially in developing countries.^[^ ^19^ ^]^ This is obviously the result of the mentioned high complexity of the heritage conservation field, the importance of the practitionerś skills and experience and the lack of a consensus scientific knowledge to support practice activities. One of the most difficult tasks that practitioners face in their activities is the identification of decay patterns and the decision about what type of protective treatment should be applied based on the huge complex context (type of substrate, material's properties, decay processes involved, and compatible products), which requires a comprehensive study. However, in practical cases, decisions about interventions are usually left to the last possible moment and sometimes they are made without a thorough study.^[^ ^62^ ^]^ In this context, there is an urgent need to develop new strategies to organize, summarize, and disseminate existing knowledge that could assist practitioners (conservation encyclopedia) during their decision‐making process on the field.

## 3 The Possible Way Forward: A Paradigm Shift to Overcome Current Limitations in Heritage Conservation Based on the Commons‐Based Peer Production Model (CBPP)

CBPP is a term coined by Harvard Law School professor Yochai Benkler,^[^ ^63^ ^]^ which describes a model of socio‐economic production in which large numbers of people work cooperatively for common benefits, especially over the internet (e.g., Wikipedia). This new model has been previously described by Prof. Elinor Ostrom (Nobel Prize Winner in Economics for her analysis of economic governance, especially the commons, 2009) who claimed at her well‐known communication at Science^[^ ^64^ ^]^ that these Peer‐to‐Peer (P2P) networks were a promising strategy for addressing several contemporary professional problems as they stimulate dialogue among peers which favors consensus, connects millions of users from all over the world and creates new shared value. One of the major characteristics of these commons‐based peer production communities is its usually nonprofit scope, open‐access aim, reduced hierarchy among peers, and that participation is mostly voluntary based on the complementary professional expertise of their users who work together to create new common shared value in an ecosystem of cooperation where all can benefit from it.^[^ ^65^, ^66^ ^]^

Over the last 10 years, studies on P2P networks have enjoyed a meteoric rise.^[^ ^67^ ^]^ This new model of production seems to be a prevailing driving force in Europe and grasped already the attention of the European Commission (EC), which funded several initiatives (mostly around Culture) to study the transformative potential these communing practices might have toward the improvement of economic dynamics and working and living conditions in Europe.^[^ ^68^, ^69^, ^70^, ^71^, ^72^ ^]^ This new socio‐technological model is clearly expected to fully flourish in this following decade and is expected to create new models of production, novel forms of society, and innovative social aggregation for community shared benefits.^[^ ^64^, ^65^ ^]^

## 4 Conclusions and Outlook

### 4.1 The Transformative Potential of CBPP in Heritage Conservation

I believe that this new way of production could represent a transformative solution to go beyond our conventional working method and to solve some of the current global challenges in heritage conservation since it could allow heritage professionals, from all over the world, to organize into digital communities and cooperatively and horizontally work to create a completely new shared value. These digital communities can be specifically created to document, exchange, transmit skills; preserve unpublished remarkable conservation practice observations or organize current knowledge (e.g., Wikipedia). I specifically hypothesize that, inspired by the Wikipedia initiative, the heritage conservation community could create similar initiatives to organize current scientific knowledge in a wiki‐like conservation encyclopedia. This initiative could provide a solution to tackle the complex and macroscopical analysis of the current large and unstructured scientific knowledge. This is because of the nature of content production in these types of platforms. Since content is constantly created and self‐controlled by the complementary and multidisciplinary expertise of all users, the organization and analysis of knowledge are undertaken consensually taking into consideration the input of all heritage professionals (i.e., scientists, conservators, architects, surveyors, technicians, archaeologists, curators, etc.) with very different angles in terms of professional vision (practice or science), environmental weather conditions issues, accessibility of materials or resources. This new way of production could allow evading the current “bias issues” concerning the traditional big data analysis while providing a representative vision of the global common knowledge, being also in a constant update. Additionally, considering the current accuracy of Wikipedia—Nature investigations found that Wikipedia is very close to Britannica in terms of the accuracy of its science entries^[^ ^73^ ^]^—this initiative could also provide significant levels of data quality, precision, and accuracy.

This new way of production based on peer cooperation and consensus‐driven structures can also help to mitigate the other existing global heritage challenges. For example, it can contribute to reducing the current inequalities between developed and developing countries since the access to knowledge could be less restrictive reducing the UNESCO‐called “information gap.” It can also improve the synergetic exchange of knowledge between science and practice promoting a real horizontal interdisciplinary interaction of all heritage professionals based on different geographical regions, socio‐economic environments, and environments. Additionally, possible initiatives such as this open‐source conservation encyclopedia can also help mitigate other global heritage challenges since it could contribute to creating a more participatory system to disseminate the current scientific knowledge (open‐science). These initiatives could also assist global heritage practitioners with new tools to enhance their conservation activities while increasing the capabilities and skills of other unskilled practitioners, promoting better learning opportunities for all, reducing inequality, and improving global competitiveness in skilled jobs.

Besides the direct benefits to the conservation practice global community, the successful implementation of Peer‐to‐Peer digital professional networks in the form of open‐source encyclopedia or a sharing‐information network can also represent a universal benefit for economically and socially marginalized communities in developing countries, since it could allow local communities to better self‐organize, self‐train, and self‐manage their cultural heritage; especially in areas with no Heritage Management Plan or with a serious lack of resources. This is contrary to the conventional working method within the conservation community, which has been largely criticized in the past for mostly benefiting the professional conservation community without considering its influence on local communities.^[^ ^74^, ^75^, ^76^ ^]^

This emerging socio‐professional production ecosystem is completely aligned with the fundamental values and main goals of the UN Agenda2030 for Sustainable Development in terms of promoting democratization, open science, open‐access learning opportunities, productive work, equitable quality interdisciplinary, assist with economic development, and also reducing inequalities within and among countries (Goals 4, 8, 10, and 17).

I believe that these emerging socio‐technological networks can serve our cultural heritage conservation professional field as innovative strategies to transform the professional field. It could provide a better, wider, and more equal access to knowledge while assisting global scientists and practitioners with new tools (e.g., a conservation encyclopedia created by its own community that professionals can check during conservation decision processes), which could truly revolutionize how heritage conservation professionals currently face heritage interventions; for the benefit of global conservation standards.

## Conflict of Interest

The author declares no conflict of interest.

## References

[1] ICOMOS , Carta del Restauro, the First International Congress of Architects and Technicians of Historic Monuments, Athens, 1931, https://www.icomos.org/en/167-the-athens-charter-for-the-restoration-of-historic-monuments (accessed: June 2021).

[2] ICOMOS , The Venice Charter, International Charter for the Conservation and Restoration of Monuments and Sites, 1964, https://www.icomos.org/charters/venice_e.pdf (accessed: April 2021).

[3] Travel & Tourism , World Economic Impact Report, World Travel & Tourism Council, London 2019.

[4] EPRS (European Parliamentary Research Service) , Cultural Heritage in EU policies PE 621.876, June 2018, https://www.europarl.europa.eu/RegData/etudes/BRIE/2018/621876/EPRS_BRI(2018)621876_EN.pdf (accessed: April 2021).

[5] R. Ahluwalia , Global Tourist Attractions Facing Extinction, From Machu Picchu to the Taj Mahal, the Independent, 2017, https://www.independent.co.uk/travel/news-and-advice/extinct-tourist-attractions-climate-change-erosion-taj-mahal-machu-picchu-mont-blanc-great-wall-china-a7809926.html (accessed: March 2021).

[6] S. Ravil , Twilight of the Taj, BBC, 2019, https://www.bbc.co.uk/news/resources/idt-sh/twilight_of_the_taj (accessed: March 2021).

[7] T. R. Paradise , Tourism and Architectural Heritage Management in Petra, Jordan (Ed: D. Comer ), Springer, New York 2011, Ch. 3.

[8] F. Chen , H. Guo , N. Ishwaran , J. Liu , X. Wang , W. Zhou , P. Tang , Remote Sens. Environ. 2019, 232, 111293.

[9] Global Heritage Fund , Saving Our Global Heritage for Future Generations, Biennial Report, Global Heritage Fund, Palo Alto, CA 2020.

[10] ICOMOS , Heritage at Risk World Report on Monuments and Sites in Danger (Eds: C. Machat , J. Ziesemer ), Hendrik Bäßler Verlag, Berlin 2020.

[11] UNESCO and UN Environment Program (UNEP) , World Heritage and Tourism in a Changing Climate, UNESCO, Paris 2016.

[12] Tourism and Culture Synergies , The World Tourism Organization, UNWTO Publications, Madrid 2018.

[13] G. Terrill , Int. J. Heritage Stud. 2008, 14, 388.

[14] UNESCO World Heritage Centre , Policy Document on the Impacts of Climate Change on World Heritage Properties, UNESCO World Heritage Centre, Paris 2008.

[15] H. Phillips , Hist. Environ.: Policy Pract. 2014, 5, 288.

[16] S. Fatorić , E. Seekamp , Clim. Change 2017, 142, 227.

[17] L. Reimann , A. T. Vafeidis , S. Brown , J. Hinkel , R. S. J. Tol , Nature Commun. 2018, 9, 4161. 30327459 10.1038/s41467-018-06645-9 PMC6191433

[18] J. Fidler , Construction Research and Innovation in Heritage Sector: Foresight Planning for a Research Strategy for the Construction Industry, English Heritage, Swindon, UK 2005.

[19] T. Kayan , Conservation of Heritage Buildings: Maintaining Old Government Building in Kaula Lumpur after Gazetted Period, University of Malaya, Kuala Lumpur 2003.

[20] M. Ripp , M. Göttler , Community Involvement in Heritage Management: Guidebook, Organization of World Heritage Cities (OWHC), Regensburg, Germany 2017.

[21] K. Van Balen , Community Involvement in Heritage, Reflections on Cultural Heritage Theories and Practices, Garant Publishers, Chicago, IL 2015.

[22] A. Imhausen , in The Mathematics of Egypt, Mesopotamia, China, India, and Islam: A Sourcebook (Ed: V. J. Katz ), Princeton University Press, Princeton, NJ 2007.

[23] S. Sagiroglu , D. Sinanc , in 2013 Int. Conf. on Collaboration Technologies and Systems (CTS), IEEE, Piscataway 2013, pp. 42–47, 10.1109/CTS.2013.6567202.

[24] A. Labrinidis , H. V. Jagadish , Proc. VLDB Endow. 2012, 5, 2032.

[25] T. Davenport , P. Barth , R. Bean , MIT Sloan Manage. Rev. 2012, 54, 1.

[26] M. Faraday , First Principles of Electricity, Royal Institution Christmas Lectures, The Royal Institution of Great Britain, London 1843.

[27] M. Gilberg , J. Am. Inst. Conserv. 1987, 26, 105.

[28] G. Wheeler , Alkoxysilanes and the Consolidation of Stone, Research in Conservation, Getty Conservation Institute, Los Angeles, CA 2005.

[29] N. Przulj , Integrated Connectedness for a New Representation of Biology (ICON‐BIO), 2018, ERC Starting Grant, https://cordis.europa.eu/project/id/770827 (accessed: April 2021).

[30] D. R. Sanz , Social Networks of the Past: Mapping Hispanic and Lusophone Literary Modernity, 1898–1959 (MapModern), 2018, ERC Starting Grant (grant agreement 803860), https://cordis.europa.eu/project/id/803860 (accessed: April 2021).

[31] S. Ibaraki , Artificial Intelligence For Good: Preserving Our Cultural Heritage, Forbes, Jersey City, NJ 2019, pp. 45–52.

[32] C. Wang , Int. J. Big Data Anal. Healthcare 2019, 4, 1.

[33] L. Cai , Y. Zhu , Data Sci. J. 2015, 14, 2.

[34] U. Sivarajah , M. M. Kamal , Z. Irani , V. Weerakkody , J. Bus. Res. 2017, 70, 263.

[35] S. Ren , Y. Zhang , Y. Liu , T. Sakao , D. Huisingh , C. Almeida , J. Cleaner Prod. 2019, 210, 1343.

[36] L. Zhou , S. Pan , J. Wang , A. V. Vasilakos , Neurocomputing 2017, 237, 350.

[37] P. Glauner , P. Valtchev , R. State , in 26th European Symp. on Artificial Neural Networks, Computational Intelligence and Machine Learning (ESANN), Bruges, Belgium, 25–27 April. ISBN 978‐287587047‐6. 2018.

[38] A. Ajšić , Language and Ethnonationalism in Contemporary West Central Balkans, Palgrave Macmillan, Cham 2021.

[39] A. Saxena , S. Chandra , Artificial Intelligence and Machine Learning in Healthcare, Springer, Singapore 2021.

[40] T. Sunderland , J. Sunderland‐Groves , P. Shanley , B. Campbell , Biotropica 2009, 41, 549.

[41] A. E. Wilson , Bioscience 2007, 57, 550.

[42] J. Coloma , E. Harris , PLoS Pathog 2005, 1, 9901. 10.1371/journal.ppat.0010021 PMC1266310 16254599

[43] D. Menefee , Publishers Commit to Bringing Free Access to Scientific Research to Developing World, Elsevier, Washington, DC 2007.

[44] D. A. King , Nature 2004, 430, 311. 15254529 10.1038/430311a

[45] C. J. Kennedy , Hist. Environ.: Policy Pract. 2015, 6, 214.

[46] A. Golfomitsou , Conservation Science, Papers arising from the ICCROM Forum on Conservation Science, ICCROM, Rome 2013.

[47] J. C. Wells , Human‐Centered Built Environment Heritage Preservation, Routledge, New York 2018.

[48] R. Mazzeo , M. Tabasso , in University Postgraduate Curricula for Conservation Scientists: Proc. of the Int. Seminar (Ed: C. Rockwell ), ICCROM, Rome 2000, pp. 1–12.

[49] B. Appelbaum , Conservation Treatment and the Custodian/Conservator Relationship, CeROART Regards contemporaries sur la restauration, 10.4000/ceroart.445 (accessed: April 2008).

[50] J. H. Stoner , Scientific Examination of Art: Modern Techniques in Conservation and Analysis, Proceedings of the National Academy of Sciences, The National Academies Press, Washington, DC 2005, p. 41.

[51] S. Muñoz‐Viñas , Contemporary Theory of Conservation, Elsevier/Butterworth Heinemann, London 2005, p. 185.

[52] Aga Khan for Culture, Aga Khan Foundation , Mughal Tilework: A Craft Revival and Conservation, Mapin Publishing, Ahmedabad, India 2020.

[53] The Aga Khan Historic Cities Programme , Strategies for Urban Regeneration, The Earthen Architecture Programme 2015, https://s3.us-east-1.amazonaws.com/media.archnet.org/system/publications/contents/6716/original/DPC3571.pdf?1384801236 (accessed: March 2021).

[54] M. S. Gill , T. H. Rehren , I. Freestone , J. Archaeol. Sci. 2014, 49, 546.

[55] UNESCO , Improving Access to Scientific Information for Developing Countries: UK Learned Societies and Journal Access Programmes, UK National Commission for UNESCO, London, http://www.unesco.org.uk (accessed: March 2021).

[56] C. A. Woolston , Nature 2015, 525, 429. 10.1038/nj7569-413a 26397002

[57] J. Otero , A. E. Charola , C. A. Grissom , V. Starinieri , GE‐Conservacion 2017, 1, 71.

[58] C. Rodriguez‐Navarro , E. Ruiz‐Agudo , Pure Appl. Chem. 2017, 90, 523.

[59] J. Otero , V. Starinieri , A. E. Charola , G. Taglieri , Constr. Build. Mater. 2020, 230, 117112.

[60] J. Otero , V. Starinieri , A. E. Charola , Constr. Build. Mater. 2018, 181, 394.

[61] J. Otero , V. Starinieri , A. E. Charola , Constr. Build. Mater. 2019, 209, 701.

[62] J. Otero , A. E. Charola , in Proc. of the 14th Int. Congress on the Deterioration and Conservation of Stone (Eds: S. Siegesmund , B. Middendorf ), Vol. I, Mitteldeutscher Verlag, Gottingen, Germany 2020.

[63] Y. Benkler , The Wealth of Networks: How Social Production Transforms Markets and Freedom, Yale University Press, New Haven, CT 2006,.

[64] T. Dietz , E. Ostrom , P. C. Stern , Science 2003, 302, 1907. 14671286 10.1126/science.1091015

[65] D. Jemielniak , A. Przegalinska , Collaborative Society, MIT Press, Cambridge, MA 2020.

[66] E. Petersen , J. Pearce , Technologies 2017, 5, 7.

[67] J. H. Reichman , P. F. Uhlir , T. Dedeurwaerdere , Global Intellectual Property Strategies for a Redesigned Microbial Research Commons, Cambridge University Press, Cambridge 2016.

[68] M. F. De Tulio , Commons. Between Dreams and Reality, Creative Industry Kosice, Košice, Slovakia 2020.

[69] L. Volont , Shapeshifting: The Cultural Production of Common Space, University of Antwerp, Antwerp, Belgium 2017, https://ccqo.eu/2020/02/21/aart-van-der-maas-culture-commons-and-community-cultural-centres-towards-an-inclusive-city/ (accessed: March 2021).

[70] M. F. De Tullio , Cultural and Creative Spaces and Cities, University of Antwerp, Antwerp, Belgium 2018, http://ccqo.eu/2018/11/29/maria-francesca-de-tullio-cultural-and-creative-spaces-and-cities/

[71] T. Van Der Maas , Culture Commons and Community Cultural Centres towards an Inclusive City, University of Applied Sciences Utrecht, Utrecht, The Netherlands 2020, http://ccqo.eu/2020/02/21/aart-van-der-maas-culture-commons-and-community-cultural-centres-towards-an-inclusive-city/.

[72] M. Bauwens , V. Kostakis , A. Pazaitis , Peer to Peer: The Commons Manifesto, University of Westminster Press, London 2019.

[73] J. Giles , Nature 2005, 438, 900. 16355180 10.1038/438900a

[74] R. Harrison , Heritage: Critical Approaches, Routledge, New York 2013.

[75] L. Smith , Uses of Heritage, Routledge, New York 2007.

[76] J. C. Wells , in Human‐Centered Built Environment Heritage Preservation: Theory and Evidence‐Based Practice (Eds: J. C. Wells , B. L. Stiefel ), Routledge, New York 2019, pp. 33–44.

